# Exogenous Sucrose Improves the Vigor of Aged Safflower Seeds by Mediating Fatty Acid Metabolism and Glycometabolism

**DOI:** 10.3390/plants14152301

**Published:** 2025-07-25

**Authors:** Tang Lv, Lin Zhong, Juan Li, Cuiping Chen, Bin Xian, Tao Zhou, Chaoxiang Ren, Jiang Chen, Jin Pei, Jie Yan

**Affiliations:** 1 State Key Laboratory of Southwestern Chinese Medicine Resources, Chengdu University of Traditional Chinese Medicine, Chengdu 611137, China; tanglv2024@163.com (T.L.); lil2237172215@163.com (L.Z.); juanli@stu.cdutcm.edu.cn (J.L.); chencuiping@cdutcm.edu.cn (C.C.); xianbin@stu.cdutcm.edu.cn (B.X.); zhoutao@cdutcm.edu.cn (T.Z.); chaoxiangren@cdutcm.edu.cn (C.R.); janshen1986@163.com (J.C.); 2 College of Pharmacy, Chengdu University of Traditional Chinese Medicine, Chengdu 611137, China

**Keywords:** aged safflower seed, exogenous sucrose, differentially expression genes, glycometabolism, germination, controlled deterioration treatment

## Abstract

Safflower (*Carthamus tinctorius* L.) seeds, rich in triacylglycerols, have poor fatty acid-to-sugar conversion during storage, affecting longevity and vigor. Previous experiments have shown that the aging of safflower seeds is mainly related to the impairment of energy metabolism pathways such as glycolysis, fatty acid degradation, and the tricarboxylic acid cycle. The treatment with exogenous sucrose can partially promote the germination of aged seeds. However, the specific pathways through which exogenous sucrose promotes the germination of aged safflower seeds have not yet been elucidated. This study aimed to explore the molecular mechanism by which exogenous sucrose enhances the vitality of aged seeds. Phenotypically, it promoted germination and seedling establishment in CDT-aged seeds but not in unaged ones. Biochemical analyses revealed increased soluble sugars and fatty acids in aged seeds with sucrose treatment. Enzyme activity and transcriptome sequencing showed up-regulation of key enzymes and genes in related metabolic pathways in aged seeds, not in unaged ones. qPCR confirmed up-regulation of genes for triacylglycerol and fatty acid-to-sugar conversion. Transmission electron microscopy showed a stronger connection between the glyoxylate recycler and oil bodies, accelerating oil body degradation. In conclusion, our research shows that exogenous sucrose promotes aged safflower seed germination by facilitating triacylglycerol hydrolysis, fatty acid conversion, and glycometabolism, rather than simply serving as a source of energy to supplement the energy deficiency of aged seeds. These findings offer practical insights for aged seeds, especially offering an effective solution to the aging problem of seeds with high oil content.

## 1. Introduction

The safflower (*Carthamus tinctorius* L.) is a versatile economic crop that grows around the world in Asia, Africa, the Americas, and Europe [1]. In 2020, the global safflower growing was about 0.8 million hectares [2]. For its reproduction, safflower depends mainly on seed, which is also the choice for production cultivation. Its seeds are one of the most important oil seeds in the world (with an oil content of approximately 23–36%), and are an important source of edible oil because of their richness in oleic and linoleic acid, especially linoleic acid (accounting for approximately 55–77% of total fatty acids), which ranks first in the oil crop kingdom [3,4,5]. However, the richness in unsaturated fatty acids makes seeds susceptible to peroxidation, leading to seed deterioration during storage and reducing germination and seedling establishment [6]. Therefore, it is feasible to explore a method to improve safflower seed vigor and seedling establishment.

Seed germination is defined as beginning with the absorption of water from a resting dry seed and ending with the protrusion of the radicle [7]. The germination progress can be divided into three phases [8]. In the first phase (imbibition), the seeds rapidly absorb water, which is a reversible physical process. The second stage is referred to as the lag phase. During this stage, the moisture content tends to stabilize, and metabolic activities intensify. The radicle breaks through the seed coat due to cell swelling, and, simultaneously, reserve substances such as oligosaccharides start to be mobilized to a limited extent. In the third stage, there is a resurgence in water absorption. The stored polysaccharides, proteins, and other key nutrients undergo extensive decomposition and are effectively utilized to facilitate the establishment of the seedlings [8,9]. Successful seed germination and seedling establishment are decisive factors in plant reproduction [10]. Usually, seeds during the germination period are susceptible to abiotic stress (e.g., salt stress [11], drought stress [12], flooding [13], and heavy metal contamination [14]) and biotic stress (e.g., fungus, insects, and weeds [15]), resulting in reduced germination and lower seedling establishment. Furthermore, seed germination is a complex process controlled by multiple endogenous and exogenous factors, including energy metabolism, hormone regulation, and gene expression regulation [16]. Together, these factors affect seed storage longevity and seed germination through different dimensions. Therefore, it is essential to reveal the physiological and molecular changes in the seed germination process.

Seed germination and seedling establishment are driven by energy in the seed itself. Before seedling establishment, seed metabolism is very low, and mainly sugars are used to produce the required energy [17]. Soluble sugars are eventually catabolized and metabolized to ATP, which is the most direct form of energy for seed germination and seedling establishment [18]. However, in oil seeds with low soluble sugar content compared to cereal crop seeds, triacylglycerol (TAG) is an efficient source of energy during oilseed germination [19]. The hydrolysis of triacylglycerols produces fatty acids (FAs) and glycerol, and FAs are converted via the β-oxidation pathway to produce acetyl coenzyme A, which forms oxaloacetate via the tricarboxylic acid or glyoxylate cycle (GAC), and then converted to sucrose via gluconeogenesis (GNG) to support seed germination and seedling establishment [20,21]. Therefore, the efficient conversion of fatty acids from triacylglycerol catabolism to sucrose is critical for oilseed seed germination. For example, impaired fatty acid catabolism to soluble sugars during early germination of aged soybean, sunflower, and safflower seeds is the principal cause for the decline in vigor of seed storage [17,22,23,24]. In addition, the degradation of structural lipids (PC, PE, PS, PI, PG) during the aging process of safflower seeds ultimately destroys the cell membrane structure, leading to a decrease in seed viability during natural seed aging [25].

In this way, scholars focus on methods to improve germination and seedling establishment in aged crop seeds. Sucrose is derived from plant photosynthesis and is critical for integrating the functions of internal and external regulatory signals that drive various physiological processes from embryogenesis to aging [26]. Meanwhile, sucrose is a substrate for the formation of storage reserves and is a major nutrient and energy source for plant growth and development [27]. The role of exogenous sucrose in promoting seed germination in crops has been reported in soybean [28], wheat [29], and Arabidopsis [30]. Exogenous sucrose may affect seed germination capacity mainly by regulating lipid metabolism [31], endogenous phytohormone homeostasis [32], redox homeostasis [33], and cell cycle [34]. In addition, exogenous sucrose can influence fatty acid catabolism and promote seed germination by regulating the glyoxylate cyclosome [35]. Furthermore, our previous report showed that exogenous sucrose was able to increase the germination rate of artificially aged safflower seeds [24]. However, this study focused only on germination and did not address seedling establishment. More importantly, the mechanism by which exogenous sucrose enhances seed viability in aging crops needs to be further investigated.

Here, we found that exogenous sucrose increased the viability of aged safflower seeds by regulating fatty acids and glycometabolism. Physiological and biochemical phenotyping, ultrastructure, transcriptome sequencing and qPCR analyses indicated that exogenous sucrose application promotes triacylglycerol hydrolysis and enhances the conversion of fatty acids to sugars, which in turn improves the viability and seedling emergence of aged safflower seeds. This study may provide a reference for the potential application of sucrose in agricultural systems and the regulatory mechanism of exogenous sucrose in enhancing the seed vigor of aging crops.

## 2. Results

### 2.1. CDT Remarkably Inhibited Safflower Seed Germination

**Figure 1:**
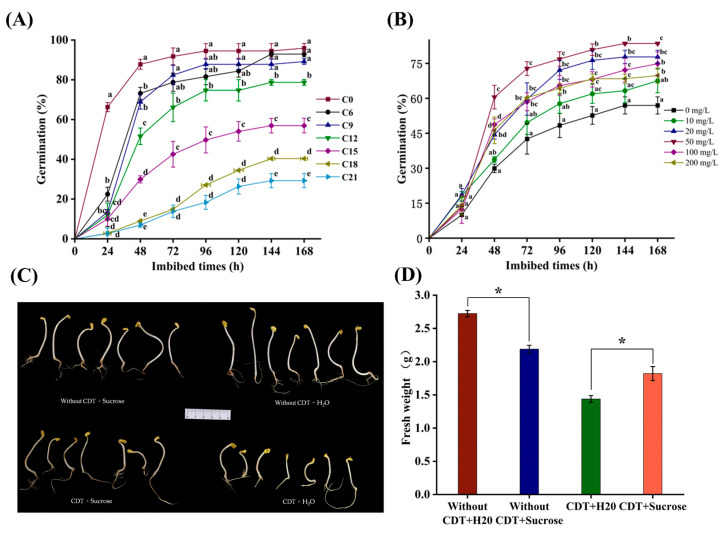
The germination conditions of seeds under different treatments (**A**) CDT significantly reduced safflower seed germination vigor. (**B**) Sucrose promotes the germination of CDT-aged 15 days safflower seeds. (**C**) Growth of seedlings with different treatments. (**D**) Fresh weight of seedlings with different treatments. Each symbol represents the average of three replicates ± SE. Different lowercase letters(s) at the top of the bars indicate significant differences between treatment types (* indicates statistically significant differences *p* < 0.05, LSD).

Safflower seed germination viability was evaluated at different CDT periods. The germination indicator decreased significantly with the increase in CDT time (Figure 1A). At the beginning of aging, the germination of C0 (day 0) and C6 (day 6) were determined as 96% and 93%, respectively. After C9 (day 9) treatment germination was 89%, and significantly lower than that of unaged (C0) seeds (Figure 1A). As aging proceeded through C12 (day 12) and C15 (day 15), germination was 79% and 57%, respectively. By C21 (day 21) germination was only 29%. Furthermore, seed germination potential and germination indexes were also reduced with CDT treatment, concomitant with decreasing germination (Figure S1A).

### 2.2. Exogenous Sucrose Treatment Promoted Germination of Aged Safflower Seeds but Not from Unaged Seeds

The effect of different exogenous sucrose concentrations on the germination of aged safflower seeds was evaluated (i.e., 0, 10, 20, 50, 100, 200 mg·L^−1^). We found that exogenous sucrose treatment improved the germination of aged safflower seeds with an increasing and then decreasing sucrose concentration (Figure 1B). In detail, the germination of aged safflower seeds was significantly increased at 20 mg·L^−1^ and 50 mg·L^−1^ sucrose concentration, with germination rates of 78% and 84%, respectively, which were 21% and 27% higher than that of the control group, while the germination was 70% at 200 mg·L^−1^ sucrose concentration, which was only 13% higher than that of the control group (Figure S1B). In addition, the germination potential and germination index showed a similar model to the germination rate, both of which were best enhanced by 50 mg·L^−1^, which increased to 60% and 30% (Figure S1B), respectively, compared with the control group.

Exogenous sucrose also had a positive effect on the establishment of seedlings from aged safflower seeds (Figure 1C). In the absence of exogenous sucrose, the fresh weights of aged safflower seeds were lower than those of unaged safflower seeds (Figure 1D). After treatment with exogenous sucrose, the growth of seedlings of aged safflower seeds was improved, as evidenced by the corresponding fresh weights (Figure 1D).

Finally, since exogenous sucrose increased the germination of aged safflower seeds, we then investigated the effect of sucrose on the germination of unaged safflower seeds. The results showed that sucrose showed no significant effect on the germination of unaged safflower seeds, but inhibited seedling growth (Figure S2). These results suggest that sucrose can only promote the germination of aged safflower seeds, but not unaged safflower seeds (Figures S1 and S2).

### 2.3. Transcriptome Analysis and Differentially Expressed Genes Between Control and Exogenous Treatments

**Figure 2:**
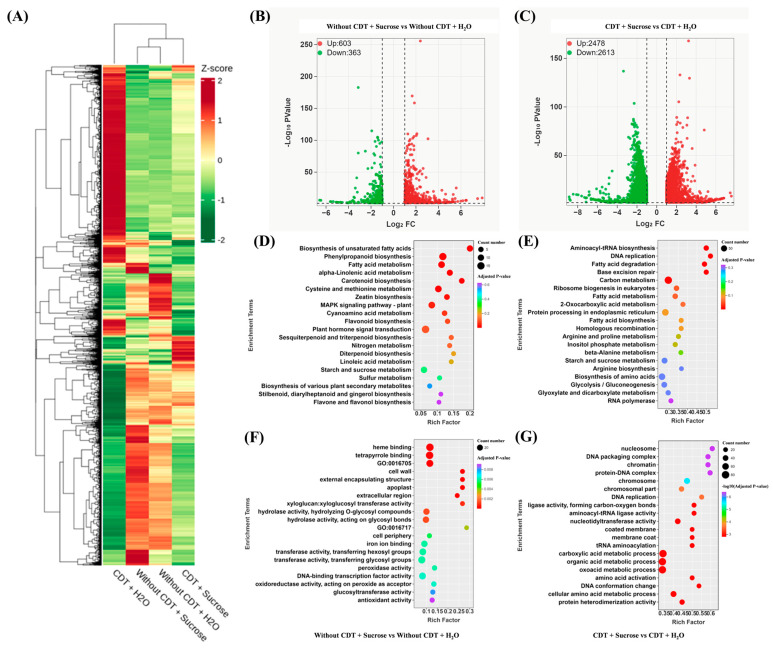
Transcriptome analysis of exogenous sucrose-treated aged safflower seeds and unaged safflower seed. (**A**) Clustered heat map analysis of CDT + Sucrose, CDT + H_2_O, Without CDT + Sucrose, and Without CDT + H_2_O. (**B**) Volcano plots of Without CDT + Sucrose vs. Without CDT + H_2_O differentially expressed genes. (**C**) Volcano plots of CDT + Sucrose vs. CDT + H_2_O differentially expressed genes. (**D**) KEGG enrichment analysis of DEGs at Without CDT + Sucrose vs. Without CDT + H_2_O. (**E**) KEGG enrichment analysis of DEGs at CDT + Sucrose vs. CDT + H_2_O. (**F**) GO enrichment analysis of DEGs at Without CDT + Sucrose vs. Without CDT + H_2_O. (**G**) GO enrichment analysis of DEGs at CDT + Sucrose vs. CDT + H_2_O.

To understand the early transcriptome changes after exogenous sucrose treatment, we collected seed samples at 12 h of imbibed and performed RNA-Seq analysis. During our transcriptome analysis, we used adjusted *p*-value < 0.05 and |Log_2_Fold Change| ≥ 1 to define differentially expressed genes (DEGs). In contrast to aged seeds, only a few genes were significantly altered between exogenous sucrose treatment and control in unaged safflower seeds (Figure 2), consistent with our expectations. The volcano plot showed that there were 2478 differentially up-regulated genes and 2613 differentially down-regulated genes in the CDT + Sucrose vs. CDT + H_2_O seed group, 603 differentially up-regulated genes and 363 differentially down-regulated genes were found in the Without CDT + Sucrose vs. Without CDT + H_2_O seed group, respectively (Figure 2B,C). The expression of a large number of genes was significantly changed under CDT + Sucrose vs. CDT + H_2_O seed conditions compared to Without CDT + Sucrose vs. Without CDT + H_2_O (Figure 2). This result implies that exogenous sucrose regulates gene expression in the early stages of germination of aged safflower seeds exceeded of unaged seeds.

Subsequently, we performed functional enrichment analysis of the differentially expressed genes using Kyoto Encyclopedia of Genes and Genomes (KEGG). KEGG enrichment analysis of CDT + Sucrose vs. CDT + H_2_O was mainly focused on primary metabolism, including glycolysis/gluconeogenesis, and arginine biosynthesis, et al. (Figure 2E), while Without CDT + Sucrose vs. Without CDT + H_2_O was mainly enriched in secondary metabolism, including flavone and flavonol biosynthesis, and phenylpropanoid biosynthesis, et al. (Figure 2D). Primary metabolisms such as sugar and lipid metabolism provide energy supply for early seed germination. Secondary metabolisms such as flavonoids and phenylpropanoids play key roles in plant reproduction, pathogen resistance or stress tolerance [36,37]. This result suggests that exogenous sucrose may enhance seed vigor by interfering with sugar and lipid metabolism in the early stage of seed germination of aged safflower seeds.

Similarly, GO enrichment analysis revealed 20 enrichment categories. In the CDT + Sucrose vs. CDT + H_2_O group, these included nucleosome, nucleotidyltransferase activity, chromosome, and aminoacyl-tRNA ligase activity, implying reprogramming of cellular metabolism after exogenous sucrose treatment (Figure 2G). Moreover, in the Without CDT + Sucrose vs. Without CDT + H_2_O group, antioxidant activity, peroxidase activity, and cell wall were enriched (Figure 2F). This shows the active cellular metabolism and transcription during safflower seed germination under exogenous sucrose treatment.

### 2.4. Exogenous Sucrose Increases the Transcription of Several Key Genes Involved in the Conversion of Triacylglycerols to Fatty Acids and Sugars During Imbibition in Aged Safflower Seeds

**Figure 3:**
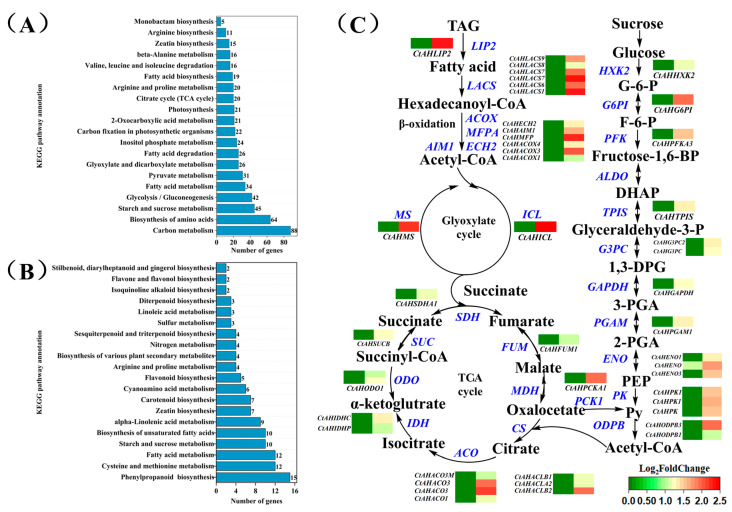
Expression of related genes in aged safflower seeds. (**A**) Expression patterns of key genes in energy metabolic pathways of aged safflower seeds. (**B**) Expression patterns of key genes in energy metabolic pathways of unaged safflower seeds. (**C**) An overview of sugar metabolism, fatty acid degradation, and TCA. Safflower seeds of treatment types (CDT seeds with H_2_O vs. CDT seeds with 50 mg·L^−1^ sucrose treatment) were employed. (Enzyme abbreviations are HXK: hexokinase; G6PI: phosphoglycerate isomerase; PFK: 6-phosphofructokinase; ALDO: fructose-bisphosphate aldolase; TPIS: triosephosphate isomerase; GAPDH: glyceraldehyde 3-phosphate dehydrogenase; PGAM: phosphoglycerate mutase; ENO: enolase; PK: pyruvate kinase; PDH: pyruvate dehydrogenase; CS: citrate synthase; ACO: aconitase; IDH: isocitrate dehydrogenase; ODO: 2-oxoglutarate dehydrogenase; SUC: succinyl-CoA synthase; SDH: succinate dehydrogenase; FUM: fumarase; MDH: malate dehydrogenase. PCK: phosphoenolpyruvate carboxykinase; MS: malate Synthase; ICL: isocitrate lyase; Py: pyruvate).

Given our previous studies, impaired fatty acid metabolism and sugar metabolism are important causes of aging in safflower seeds. Therefore, we further analyzed the effects of exogenous sucrose on the conversion of triacylglycerols to fatty acids and sugars in aged safflower seeds by studying the effects of exogenous sucrose on the expression of genes for glycolysis (EMP), fatty acid degradation, the tricarboxylic acid cycle, the glyoxylate cycle, and gluconeogenesis. The results of transcriptome analysis showed that the expression of the key gene of glycolysis, fatty acid degradation, tricarboxylic acid cycle, and glyoxylate cycle was significantly up-regulated in aged safflower seeds under exogenous sucrose treatment, whereas no significant alteration was observed in unaged safflower seeds (Figure 3A,B), e.g., *triacylglycerol lipase 2* (*LIPG2*), *isocitrate lyase* (*ICL*), *malate synthase* (*MS*), *phosphoenolpyruvate carboxykinase 1* (*PCKA1*), *Pyruvate kinase* (*PK*), etc. (Figure 3C). It indicated that exogenous sucrose treatment increased the conversion of fatty acids to soluble sugars and promoted the utilization of soluble sugars in aged safflower seeds.

### 2.5. qPCR Verification for Expressions of Exogenous Sucrose Altered Genes

**Figure 4:**
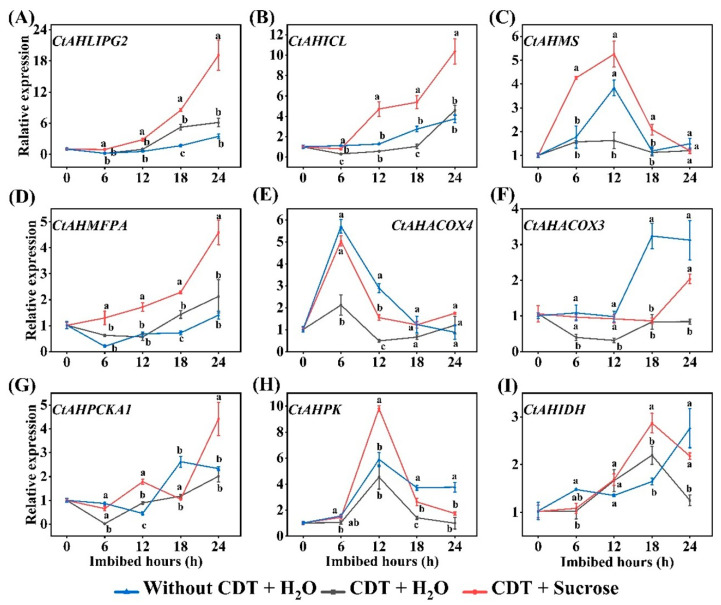
Sucrose enhances the transcript levels of several key genes involved in the conversion of fatty acids to sugars during imbibition in aged safflower seeds. Safflower seeds of treatment types (seeds without CDT, CDT seeds with H_2_O, and CDT seeds with sucrose treatment) were employed. (**A**) *CtAHLIPG2*; (**B**) *CtAHICL*; (**C**) *CtAHMS*; (**D**) *CtAHMFPA*; (**E**) *CtAHACOX3*; (**F**) *CtAHACOX4*; (**G**) *CtAHPCKA1*; (**H**) *CtAHPK*; (**I**) *CtAHIDH*. LIPG: triacylglycerol lipase; ICL: isocitrate lyase (ICL); MS: malate synthase; ACOX: acyl-CoA oxidase; PCK: phosphoenolpyruvate carboxykinase; PK: pyruvate kinase; IDH: isocitrate dehydrogenase. Each symbol represents the average of three replicates ± SE. Different lowercase letters(s) at the top of the bars indicate significant differences between treatment types (*p* < 0.05, LSD). Exogenous sucrose at 50 mg·L^−1^ was employed.

Subsequent qPCR analysis indicated transcript levels of key genes in the pathway from triacylglycerol hydrolysis to conversion to soluble sugars, namely *CtAHLIPG2*, *CtAHICL*, *CtAHMASY*, *CtAHMFPA*, *CtAHACOX3*, *CtAHACOX4*, *CtAHPCK1*, *CtAHPK*, *CtAHIDH*, were all differentially up-regulated during imbibition (0–24 h) of exogenous sucrose treatment aged safflower seeds as compared to aged seeds without exogenous sucrose treatment, especially in *CtAHLIPG2* and *CtAHICL* (Figure 4A–G). The transcript levels of *CtAHPK* and *CtAHIDH*, which are involved in glycolysis and the TCA cycle, were also increased (Figure 4H,I). In addition, the transcript levels of *CtAHICL*, *CtAHMS*, *CtAHACOX3*, *CtAHACOX4*, and *CtAHPK* were reduced in CDT-treated seeds compared with those of the without CDT (Figure 4). Altogether, this suggests that exogenous sucrose not only promotes the conversion of triacylglycerol to fatty acids and sugar but also promotes the metabolic energy supply process from sugar to the TCA cycle, which provides the energy required for aged seed germination.

### 2.6. Exogenous Sucrose Treatment Increased the Concentrations of Various Sugars in Aged Safflower Seeds During Imbibition

**Figure 5:**
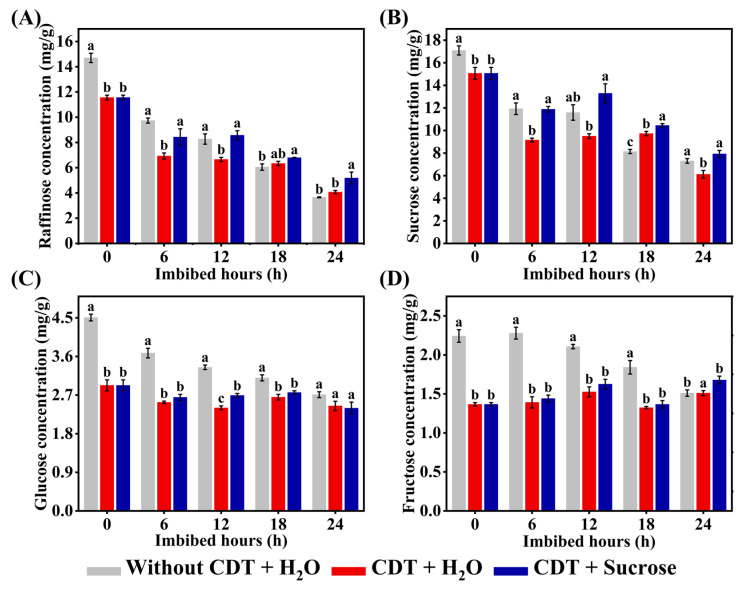
Sucrose treatment increased the concentration of soluble sugars in aged safflower seeds during imbibition. Safflower seeds of treatment types (seeds without CDT, CDT seeds with H_2_O, and CDT seeds with sucrose treatment) were employed. (**A**) The raffinose concentration of different treatment types of safflower seeds during imbibition. (**B**) The sucrose concentration of different treatment types of safflower seeds during imbibition. (**C**) The glucose concentration of different treatment types of safflower seeds during imbibition. (**D**) The fructose concentration of different treatment types of safflower seeds during imbibition. Each symbol represents the average of three replicates ± SE. Different lowercase letters(s) at the top of the bars indicate significant differences between treatment types (*p* < 0.05, LSD). Exogenous sucrose at 50 mg·L^−1^ was employed.

Seed storage substance is the main source of energy during early seed germination and seedling establishment, and sucrose, glucose, and fructose are the main nutrients produced by the breakdown of storage material [22]. Additionally, seed raffinose family oligosaccharides (RFOs) are converted to sucrose and galactose by α-galactosidase during germination [38]. Therefore, we further investigated the changes in raffinose, sucrose, glucose, and fructose concentrations during early imbibition (0–24 h). The results showed that the concentration of raffinose, sucrose, glucose, and fructose exhibited a decreasing pattern during safflower seed imbibition (Figure 5). It is noteworthy that the concentrations of raffinose and sucrose in CDT-aged safflower seeds under exogenous sucrose treatment were invariably significantly higher than those of CDT-aged seeds without exogenous sucrose treatment (Figure 5A,B). Glucose and fructose concentrations were significantly increased at 12 and 24 h of uptake respectively under exogenous sucrose treatment (Figure 5C,D). Furthermore, the concentration of sucrose, glucose, and fructose without CDT-aged safflower seeds was higher in CDT-aged safflower seeds. These results suggest that sucrose increases seed viability and seedling establishment by increasing the concentration of soluble sugars (raffinose, sucrose, glucose, and fructose) during the imbibition.

### 2.7. Exogenous Sucrose Treatment Increased the Concentrations of Fatty Acids in Aged Safflower Seeds During Imbibition Time

As is known, triacylglycerols (oil bodies) are hydrolyzed to fatty acids and glycerol during seed germination and early seedling establishment in oilseed crops, while fatty acids are important precursors for the production of soluble sugars. Therefore, we determined the concentration of several fatty acids during safflower seed imbibition.

**Figure 6:**
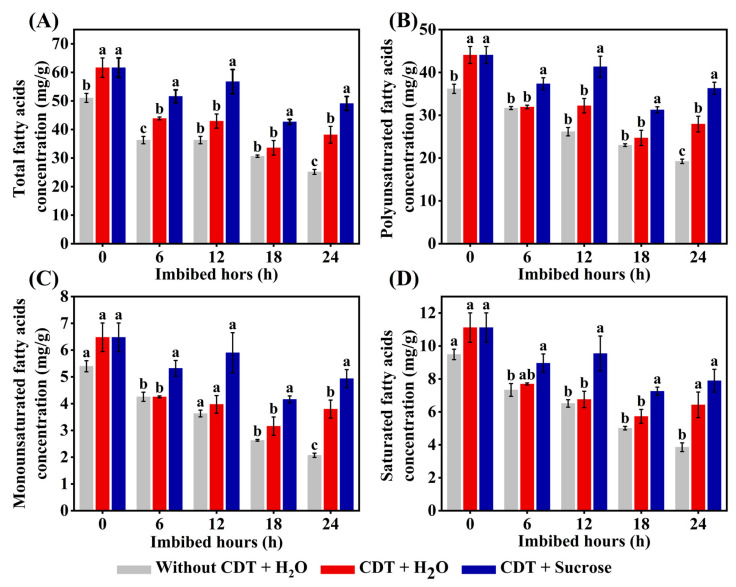
Sucrose treatment increased the concentration of fatty acids in aged safflower seeds during imbibition. Safflower seeds of treatment types (seeds without CDT, CDT seeds with H_2_O, and CDT seeds with sucrose treatment) were employed. (**A**) The total fatty acids concentration of different treatment types of safflower seeds during imbibition. (**B**) The polyunsaturated fatty acids concentration of different treatment types of safflower seeds during imbibition. (**C**) The monounsaturated fatty acids concentration of different treatment types of safflower seeds during imbibition. (**D**) The saturated fatty acids concentration of different treatment types of safflower seeds during imbibition. Each symbol represents the average of three replicates ± SE. Different lowercase letters at the top of the bars indicate significant differences between treatment types (*p* < 0.05, LSD). Exogenous sucrose at 50 mg·L^−1^ was employed.

GC-MS analysis showed that CDT treatment increased the concentration of total fatty acids, polyunsaturated fatty acids (PUFAs), monounsaturated fatty acids (MUFAs), and saturated fatty acids (SFAs) (Figure 6), which were similar to the results of previous studies. It is worth noting that the fatty acids concentration of sucrose-treated CDT-aged safflower seeds was higher than that of without sucrose treatment seeds. The concentration of total fatty acids, PUFAs, MUFAs, and SFAs in the sucrose treatment CDT-aged safflower seeds was all higher than the corresponding levels in CDT seeds in the absence of sucrose treatment (Figure 6). In addition, total fatty acid levels were increased in CDT + H_2_O seeds compared to seeds without CDT. Finally, we investigated the effect of sucrose treatment on the concentration of each of the major types of fatty acids in safflower seeds, i.e., palmitic, stearic, oleic, and linoleic (Figure S3). The results showed that sucrose treatment increased the concentrations of each fatty acid in aged safflower seeds during the imbibition.

### 2.8. Exogenous Sucrose Increased the Activity of ICL and PK in Aged Safflower Seeds During Imbibitions

**Figure 7:**
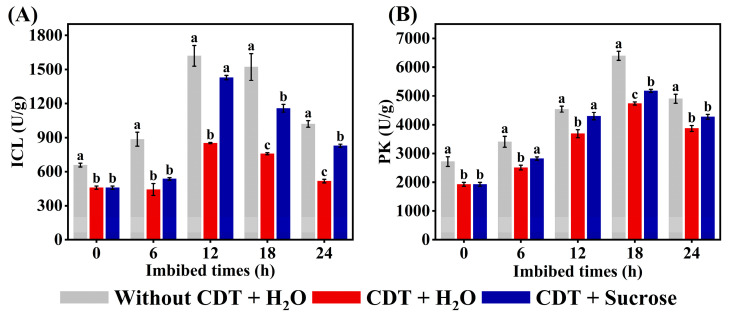
Exogenous sucrose treatment increases related enzyme activities. (**A**) Isocitrate lyase (ICL); (**B**) Pyruvate kinase (PK). Each symbol represents the average of three replicates ± SE. Different lowercase letters(s) at the top of the bars indicate significant differences between treatment types (*p* < 0.05, LSD). Exogenous sucrose at 50 mg·L^−1^ was employed.

Given that the application of exogenous sucrose increases the levels of soluble sugars and fatty acids during the imbibition of aged safflower seeds, we investigated the function of sucrose in the activity of key enzymes associated with the glyoxylate cycle and glycolysis (Figure 7). CDT treatment significantly reduced the activities of ICL, and PK in the early stage of safflower seed germination. In contrast, isocitrate lyase, and pyruvate kinase activities were significantly up-regulated in CDT+ sucrose seeds as compared to CDT + H_2_O treatments for 6, 12, 18, and 24 h. These results are consistent with the positive effect of sucrose on fatty acid and soluble sugar concentration in CDT-treated safflower seeds.

### 2.9. Changes in the Interaction of Glyoxysome with Oil Bodies Under Sucrose Treatment

**Figure 8:**
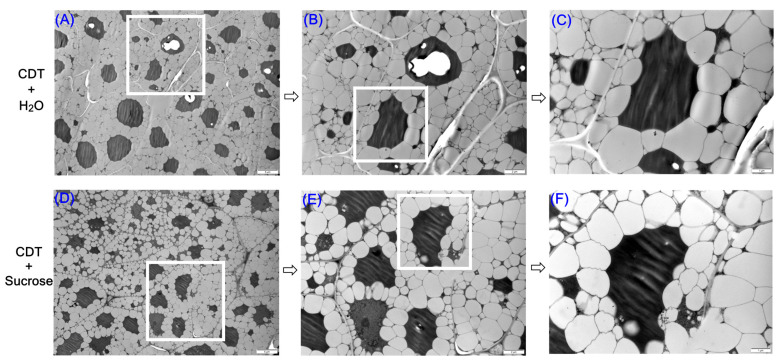
Sucrose treatment the oil bodies of aged safflower seeds to become smaller and to closely cluster near the glyoxysome. [CDT + Sucrose (**D**–**F**); CDT + H_2_O (**A**–**C**)]. In (**C**,**F**), the white part represents the oil bodies, and the black part in the middle represents the glyoxysome. The white box in the diagram indicates the location of +the next diagram enlargement. The bar is 5 μm, 2 μm, 1 μm in order.

We used transmission electron microscopy to study the ultrastructural changes between the glyoxysome and the oil body in aged safflower seeds under exogenous sucrose treatment. We found that the size of the oil bodies in aged safflower seeds decreased under exogenous sucrose treatment, and the remaining oil bodies were tightly gathered around the glyoxysome (Figure 8A,D), implying an acceleration of their degradation, as evidenced by the increased in fatty acids concentration (Figure 3). In addition, the contact surface between the oil bodies and the glyoxysome was more regular in the un-sucrose-treatment seeds, whereas the oil bodies were more concavely aggregated towards the glyoxysome under sucrose treatment (Figure 8B,C,E,F). This implies that exogenous sucrose may promote the degradation of oil bodies by facilitating the interaction between oil bodies and the glyoxysome to provide energy for early germination of aged safflower seeds, which, in turn, promotes aged safflower seed germination (Figure 1B and Figure 8).

## 3. Discussion

Seed aging is inevitable during storage. Therefore, the study of seed pretreatment methods to improve the viability of aging seeds from the perspective of energy substance metabolism and its molecular mechanisms is a worthy goal for agricultural production. In this study, phenotypic analyses, sugar concentration and fatty acid quantification, ultrastructural, and transcriptome and enzyme activity analyses indicated that sucrose partially reversed the decline in germination of aged safflower seeds. The molecular mechanism is as follows: in aged safflower seeds, exogenous sucrose promotes the hydrolysis of triacylglycerols to fatty acids and glycerol and the ability to convert fatty acids and glycerol to soluble sugars, which provide the energy required for early germination of aged safflower seeds.

### 3.1. Exogenous Sucrose Has a Positive Effect on Germination in Aged Safflower Seeds, but Not in Unaged Ones

Sucrose is not only a key component of substance metabolism but also an important signaling molecule in cellular metabolism [39]. Some studies have indicated that sucrose is engaged in the regulation of storage lipid mobilization [31]. Although the use of exogenous sucrose in seeds has been studied, this is far from sufficient, especially the molecular mechanisms. For example, exogenous sucrose promoted the rate of fatty acid catabolism in Arabidopsis thaliana seeds with impaired glyoxylate cycling to rescue their seedling growth [30]. And exogenous sucrose affected plant growth and development by regulating the activity and gene expression of the key enzymes of the glyoxylate cycle, isocitrate lyase and malate synthase, in germinating seeds and seedlings of yellow lupin [31]. Meanwhile, sucrose can improve the antioxidant capacity of maize under salt stress and promotes seed germination [33]. In addition, exogenous sucrose treatment promoted the accumulation of cytokinins, as well as the expression of vesicle-transforming enzymes to promote the branching of potato stems [40]. This study extends the practical application of sucrose by showing that exogenous sucrose can reverse the loss of aged-associated germination activity in safflower seeds, providing a reference for improving seed vigor.

However, exogenous sucrose treatment had no significant effect on unaged safflower seeds (Figure S2). In aged seeds, the positive effect of sucrose likely stems from its role compensating for oxidative damage-induced energy deficits. Aged treatments promote lipid peroxidation and protein carbonylation [41], which impair mitochondrial function and reduce ATP production via TCA cycle. Exogenous sucrose may serve as an alternative carbon source, bypassing compromised β-oxidation of stored lipids and directly fueling the TCA cycle to restore ATP levels. This is consistent with studies showing that sugars alleviate oxidative stress by supporting proteasomal activity and de novo protein synthesis during germination [42]. In contrast, unaged seeds exhibit intact antioxidant systems and efficient mobilization of stored lipids, providing sufficient energy for germination without exogenous sucrose. Therefore, although we proposed a new pathway, where exogenous sucrose, acting as a signaling molecule rather than an energy source, upregulated the related genes on the fatty acid metabolism-TCA cycle axis (especially for the ICL and PK of the glyoxylate cycle), thereby promoting seed germination, the reason why exogenous sucrose can effect the germination of aged seeds but has no effect on unaged seeds is that unaged seeds possess a well-developed antioxidant system and do not require exogenous sucrose as a signaling molecule or/and energy source to promote TCA cycle.

Moreover, physical, chemical, and biological methods have been extensively investigated in enhancing the seed vigor of aging crops. Previous studies have shown that ultrasound treatment promotes germination and seedling establishment in aging soybean seeds by increasing antioxidant defenses and regulating gluconeogenesis [43]. In addition, novel hydrated graphene ribbons promoted the germination of stored wheat seeds and improved seedling establishment by increasing soluble sugar, FAs, and amino acid content, as well as enhancing cell membrane integrity [44]. In recent years, nano-priming has emerged as an innovative seed-priming technology that contributes to a more effective approach to improving seed germination, growth, and yield by providing the ability to respond to various plant stresses [45], such as ZnO NPs [46], Fe_3_O_4_ NPs [47], and so on. The above studies have shown that seed vigor and seedling establishment can be enhanced by suitable methods which can be of great benefit to agricultural production.

### 3.2. Exogenous Sucrose Promoted the Hydrolysis of Triacylglycerol to Sugars in Aged Safflower Seeds

In the case of oil seeds, triacylglycerol lipase (LIPG) hydrolyzes triacylglycerols to produce FAs and glycerol [19,48]. We found that fatty acid concentration and *CtAHLIPG2* expression were elevated under exogenous sucrose treatment (Figure 4), suggesting that exogenous sucrose treatment promoted triacylglycerol degradation, as evidenced by transmission electron microscopy results (Figure 8). The fatty acids are catabolized via the β-oxidation pathway to acyl-CoA to enter the glyoxylate cycle [49]. ACOX, MFPA, ECH2, and AIM1 is involved in the β-oxidation pathway [50,51,52,53]. Arabidopsis seedlings of the double mutants *acox1-1 acox2-1* and mutant *mfp2-1* are unable to catabolize storage lipids for seedling establishment, which is rescued by treatment with sucrose [54,55]. In this study, these genes involved in β-oxidation in aged safflower seeds were significantly up-regulated under exogenous sucrose treatment (Figure 2 and Figure 4), supplying more raw material for the glyoxylate cycle to be converted to sugar, a result that is further evidenced by increased raffinose, sucrose, glucose, and fructose levels.

Generally, there is a positive correlation between ATP content and germination and seedling establishment [56]. The main source of energy used during seed germination and seedling establishment is soluble sugars, mainly sucrose and fructose [57]. Numerous studies have shown that changes in sugar concentration are closely related to seed vigor and longevity [28,58,59]. During the accelerated aging of maize seeds, the decrease in seed vigor was positively correlated with a significant decrease in the concentrations of monosaccharides raffinose [60]. In soybeans, increased RFO-to-sucrose ratios are positively correlated with seed maturity, viability, and longevity [61]. In addition, sucrose is a substrate for the formation of storage reserves, and during germination sucrose is mainly hydrolyzed to produce glucose and fructose, which provide energy substances and precursors for seed germination [62]. In this study, it was found that the concentrations of raffinose, sucrose, glucose, and fructose were increased in the early stage of seed germination after treatment with exogenous sucrose (Figure 5), which indicated that the direct energy substances and precursors for ATP production in aging safflower seeds were supplemented under sucrose treatment, which promoted seed germination (Figure 1B and Figure 5).

Moreover, the total fatty acids, PUFAs, MUFAs, and SFAs concentrations in safflower seeds were elevated under exogenous sucrose treatment (Figure 6). Fatty acid content regulation of seed longevity varies among species. Linoleic acid content in sweet pepper was positively correlated with germination ability, while palmitoleic acid content in seeds was negatively correlated with seed vigor [63]. However, in soybean seeds, high linoleic and linolenic acid content favors soybean seed degradation and rapid loss of vigor [64]. In orchid seeds, fatty acid degradation is a typical response to seed aged, but the link between fatty acid degradation and survival has not yet been elucidated [65]. This suggests that the variation in the content of different fatty acids may be related to the regulation of exogenous sucrose on the germination ability of aged safflower seeds, but the underlying mechanism needs to be further investigated.

### 3.3. The Glyoxysomes Plays an Important Role in the Conversion of Fatty Acids to Sugars

Glyoxysomes are specialized peroxisomes that degrade TAGs via β-oxidation and the glyoxylate cycle [57,66]. Thus, the interaction of the glyoxysomes with the oil body is critical for oil body degradation. For example, the total volume of peroxisomes increased and the transcript levels of genes encoding malate synthase, malate dehydrogenase, isocitrate lyase, and citrate synthase increased when chlamydomonas cells were grown on acetate medium [67]. Furthermore, in carrot seeds, both glyoxysomes and mitochondria were found to appear earlier in endosperm cells under hydro-electro hybrid priming, and isocitrate lyase and pyruvate kinase also improved [68]. In this study, the closer contact between the oil body and the glyoxysomes under exogenous sucrose treatment increased the degradation of the oil body (Figure 8). Interestingly, in Phalaenopsis aphrodite, contact between the oil body and the glyoxysomes was reduced by sucrose treatment [69].

During development from dry seed to seedling establishment, the glyoxylate cycle becomes active, mobilizing stored lipids [70]. Two key enzymes of the glyoxylate cycle, malate synthase and isocitrate lyase, result in the net conversion of two molecules of acyl-CoA to a single molecule of succinate. Succinate is then converted via the TCA to malate and oxaloacetate, the latter of which is synthesized into phosphoenolpyruvate via phosphoenolpyruvate carboxykinase (PCK) [70]. Phosphoenolpyruvate is converted via the gluconeogenesis pathway to sucrose, which, in turn, supports seed germination and seedling establishment [71]. Glyoxylate cycle-deficient mutants not only fail to mobilize oil bodies, but also fail to develop vesicle-like membranes and expand cotyledon cells in the light [66]. Thus, the glyoxylate cycle is regarded as essential for postemergence growth and seedling establishment in oilseed plants. However, in cereal plants, the endosperm is a main reserve of carbohydrates, and therefore the glyoxylate cycle serves other metabolic purposes than the conversion of lipids to carbohydrates [70]. In this study, exogenous sucrose significantly increased the fatty acid and soluble sugar contents of aged safflower seeds at the early stage of imbibing (Figure 5 and Figure 6). Further analysis showed that exogenous sucrose treatment increased ICL and MS activity, as well as the gene expression levels of *CtAHICL*, *CtAHMS*, and key genes for gluconeogenesis (*CtAHPCKA1*) (Figure 3, Figure 4 and Figure 7). Similarly, KIEMR treatment improves heat stress tolerance during cucumber seed germination by acting on the glyoxylate cycle [72].

In addition to determining changes in energy substance composition and concentration, changes in energy substance metabolism were also observed under exogenous sucrose treatments. The glycolysis pathway and the tricarboxylic acid cycle (TCA) are activated to provide material and energy for subsequent seed germination and seedling growth and development [73]. Glycolysis catabolizes metabolized glucose to provide pyruvate for the TCA and mitochondrial respiration for further ATP production. Pyruvate kinase and phosphofructokinase (PFK) are important rate-limiting sites in the glycolysis pathway because they catalyze two irreversible steps [74]. Studies have shown that deficiency of pyruvate kinase and disruption of function will cause seed glucose accumulation, and decreased energy levels, leading to impaired seed germination and seedling establishment [75]. In this experiment, the expression levels of most of the genes involved in encoding glycolysis and the tricarboxylic acid cycle were significantly increased (Figure 3), as evidenced by the increase in pyruvate kinase (Figure 7B).

### 3.4. Perspectives Research on Seed Vigor Repair Mechanisms

Presently, the mechanisms for increased vigor in aging seeds revolve around energy substance metabolism, redox balance, and hormone regulation [76,77,78]. However. the enhancement of seed viability and successful initiation are equally dependent on DNA repair mechanisms activated during imbibition [79]. Our transcriptome analysis showed that DNA replication and base excision repair under exogenous sucrose treatment were significantly enriched in KEGG (Figure 2), and the expression levels of related genes were significantly higher than those treated with sucrose (Table S1). Some studies have been conducted to investigate DNA damage and repair during seed vigor enhancement. Controlled rehydration with sodium chloride reduces DNA damage, reactivates antioxidant and DNA repair mechanisms, and promotes cell cycle progression, thereby facilitating seed germination [80]. Hydropriming and biopriming upregulated the expression of DNA damage repair genes, which, in turn, promoted alfalfa seed germination [81]. Therefore, the relationship between sucrose and DNA damage and repair signaling pathways during germination of aged safflower seeds needs to be further investigated. For example, does exogenous sucrose regulate nucleic acid (DNA and RNA) biosynthesis and/or signaling pathways? How does exogenous sucrose mediate DNA damage repair pathways? Furthermore, whether exogenous sucrose can regulate germination and early seedling establishment in aged seeds of other crops (e.g., soybean, wheat, rice, and maize) is also worth exploring.

## 4. Materials and Methods

### 4.1. Plant Materials

Seeds of safflower (*Carthamus tinctorius* L.) were collected from Kunming of Yunnan Province, China, in June 2022. The seeds (actually follicles) of the safflower are oblong or wedge-shaped, slightly flattened, measuring 5 to 8 mm in length and 3 to 5 mm in width. The surface is smooth or has fine wrinkles. Usually, there are 4 longitudinal ridges. When mature, they are white, grayish-white or light brown, with some dark lines or spots. The top is slightly flat or slightly concave, without awns, and the base tapers gradually. The fruit skin is hard and waxy, containing a membranous seed coat that protects the internal embryo. The seeds are of non-nutritious type and have two thick cotyledons (Figure S5). The collected seeds were dried under natural conditions. The original germination level was 98%. Before the experiments, the seeds were held under medium-term storage conditions of <45% RH and 4 °C in sealed containers.

### 4.2. Controlled Deterioration Treatment

Before the controlled deterioration treatment (CDT), the seeds were surface sterilized by immersion in 5% sodium hypochlorite solution (Solarbio, Beijing, China) for 5 min and then rinsed three times with distilled water. The assay of CDT was performed according to the protocol described by [23]. Briefly, the safflower seed samples were placed in a seed aging chamber and incubated at 50 °C and 60% RH for 0, 6, 9, 12, 15, 18, and 21 d. Then, the processed seeds were dried to their original weight at ambient temperature and then subjected to germination tests, exogenous sucrose, ultrastructural observation physiological and biochemical analyses, and transcriptome sequencing.

### 4.3. Germination Test and Seeding Establishment

After the seeds were sterilized by immersion in 5% sodium hypochlorite solution as described above, the seeds were incubated on different concentrations of agar-sucrose medium (Solarbio, Beijing, China) and 1% agar-water medium (Solarbio, Beijing, China), respectively, at 25 °C under dark conditions. Twenty-five seeds were placed in each Petri dish and three replicates for each germination assessment. Seed germination was recorded daily during the 7 days of germination. Seed germination is defined as radicle emergence by >2 mm [82]. After seed germination, 7 seedlings were randomly selected for each treatment to weigh fresh weight and repeated three times. The parameters of germination that were determined were:

Germination rate (%) = (number of germinated seeds by 7 day/total number of test seeds) × 100%

Germination potential (%) = (number of germinated seeds by 2 days/total number of test seeds) × 100%

Germination index = ∑(Gi/Ti), where Gi is the number of seeds that germinated, and Ti is the day of the germination test

### 4.4. Determination of Various Sugar Concentration

The sugar concentration in safflower seeds was determined according to a published method [83]. Samples of different imbibed times were taken and made into lyophilized powder (i.e., 0, 6, 12, 18, and 24 h). About 200 mg samples were put into a 10 mL scaled centrifuge tube with the addition of 5 mL of 80% ethanol solution and placed in an 80 °C water bath for 10 min, shaken three to five times in the process. Next, the supernatant was collected by centrifugation at 5000× *g* for 10 min. The above operation was repeated and the supernatants were combined. The extracts were filtered through a 0.22 μm organic phase filter (Jinteng, Tianjin, China). Sugar content was determined by direct HPLC measurements using a water binary HPLC system (Waters, Milford, MA, USA) equipped with a refractive index detector (Waters, Milford, MA, USA). Analyze the conditions as follows: column Agilent Hi-Plea Ca (8% cross-linked), 300 × 7.7 mm, 8 µm in diameter (Agilent Technologies, Santa Clara, CA, USA); column temperature, 85 °C; mobile phase, Milli-Q water; and flow rate, 0.6 mL·min^−1^. Data were collected and processed by the Waters chromatography station DataApex (Prague, Czech Republic). Sugars were identified by comparison with retention times and coelution of authentic standard solutions.

### 4.5. Fatty Acid Extraction and Measurements

The fatty acids were extracted according to a published protocol [84]. In brief, safflower seeds were ground with liquid nitrogen. A total of 3 mL of n-hexane (100 mg per tube) was added to the grounded safflower seeds, and the samples were extracted by ultrasonic extraction (40 kHz) for 15 min and then kept at 25 °C for 3 h. The samples were then centrifuged at 6000× *g* for 10 min at 4 °C. The supernatant was mixed with 3 mL of methanolic potassium hydroxide solution (0.4 M), vortexed for 30 s, and esterification was carried out for 1 h. The sample was centrifuged again at 6000× *g* for 10 min at 4 °C. The supernatant was transferred to a 5 mL volumetric flask, hexane was added, and the final volume was adjusted to 5 mL. The extract was filtered through a syringe filter (0.22 µm), and the extract was injected into the gas chromatography-mass spectrometry (GC-MS) system (Agilent Technologies, Santa Clara, CA, USA). Afterward, the identification and quantification of each fatty acid was performed using the previously described method with minor modifications [84].

### 4.6. Measurement of Enzyme Activity

The activity of pyruvate kinase (PK), and isocitrate lyase (ICL) was determined with kits from Solarbio (ICL, Beijing, China; PK, Beijing, China). Briefly, different types of suckered seeds were taken and soaked for 12 h. The seed coat was peeled off and quickly ground with liquid nitrogen. About 0.1 g of tissue was taken and 1 mL of extract was added and homogenized in an ice bath. After centrifugation at 15,000× *g* or 8000× *g* for 20 or 10 min at 4 °C, the supernatant was taken and placed on ice for determination of isocitrate lyase, pyruvate kinase activities. The substrates and samples were accurately reacted at 25 °C, and 37 °C in a water bath for 2 min, respectively, and, finally, the changes in absorbance values before and after the reaction were recorded at 340 nm. Consumption of 1 nmol NADH or NADPH per gram of tissue per minute in the reaction system was defined as one unit of enzyme activity.

### 4.7. RNA Extraction, Sequencing, and Data Analysis

Samples for transcriptome analysis were collected directly after 12 imbibition of different types of safflower seeds (Without CDT + H_2_O, CDT + H_2_O, CDT + Sucrose). The hard seed coat was removed and the remaining tissue was frozen in liquid nitrogen. RNA was isolated from the homogenized samples and quantified using an Agilent 2100 Bioanalyzer (Santa Clara, CA, USA). The sequencing library was generated using the following steps. The starting total RNA was used to enrich mRNA with polyA tail, followed by random interruption of the obtained mRNA with divalent cations in the Fragmentation Buffer. Then, the first strand of cDNA was synthesized in the M-MuLV reverse transcriptase system using fragmented mRNA as a template and random oligonucleotides as primers. The RNA strand was then degraded using RNaseH, and the second strand of cDNA was synthesized using dNTPs in the DNA polymerase I system. The purified double-stranded cDNA was then end-repaired, A-tailed, and ligated into sequencing junctions. The cDNA was screened with AMPure XP beads for cDNAs of 370–420 bp, amplified by PCR, and the PCR products were purified using AMPure XP beads again to obtain the final library. The sequencing library was then sequenced on the NovaSeq 6000 platform (Illumina, San Diego, CA, USA) by BGI Genomics. References to genomes and annotation files to published files [85].

### 4.8. Real-Time Quantitative Polymerase Chain Reaction

Gene expression was verified using quantitative real-time polymerase chain reaction (qPCR). Total RNA was extracted from imbibition 0, 6, 12, 18, and 24 h of different types of safflower seeds (Without CDT, CDT + H_2_O, CDT + Sucrose) for using TransZol Up Plus RNA Kit (Transgen, Beijing, China). The total RNA was treated with PrimeScript™ RT reagent Kit with gDNA Eraser (TaKaRa, Kusatsu, Japan) to remove contaminating genomic DNA. The target gene and one housekeeping gene (25 s) were designed using NCBI and synthesized by DynaTech Biotech (Chengdu, China), and the primer sequences are listed in Table S2. An aliquot of 20 µL of PCR mixture consisting of 10 µL of TB Green Premix Ex Taq II (TliRNaseH Plus), 1 µL of PCR forward primer, 1 µL of PCR reverse primer, 1 µL of DNA template and RNase-free water in a total volume of 20 µL. The annealing temperature varied for different genes. The 2^−∆∆CT^ method was used to calculate the relative expression of circular RNA [86].

### 4.9. Transmission Electron Microscopy

After prefixation with 2.5% glutaraldehyde, the tissues were refixed in 1% osmium tetroxide, dehydrated step by step with acetone in different concentration gradients, after which the dehydrating agent and Epon-812 were sequentially infiltrated in different ratios, and finally encapsulated with Epon-812. Ultrathin sections of the tissue were obtained under an ultramicrotomene and stained sequentially with uranyl acetate and lead citrate. Finally, the tissue sections were examined with a JEM-1400FLASH transmission electron microscope (JEOL, Tokyo, Japan) and images were collected.

### 4.10. Statistical Analysis

The data obtained from three replicates were expressed as means ± SE. The Statistical Product and Service Solutions (SPSS 19.0) software was used for data statistical analysis by one-way analysis of variance (ANOVA) and Student’s *t*-test. The graphing software used was OriginPro (2021).

## 5. Conclusions

**Figure 9:**
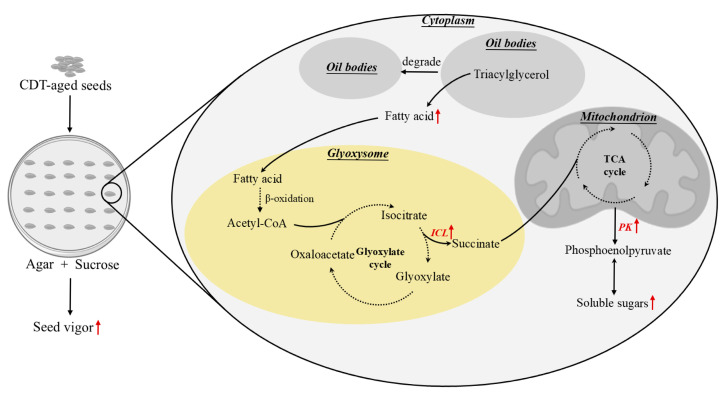
The pattern of exogenous sucrose in promoting germination in aged safflower seeds. The red up arrows indicate an increase in content or an elevation in enzyme activity.

In conclusion, this study revealed that the exogenous sucrose application could improve the germination capacities of artificially aged safflower seeds, but not from unaged safflower seed. During the imbibed process of aged safflower seeds, exogenous sucrose enhanced the hydrolysis rate of triacylglycerol into fatty acids and glycerin. At the same time, it promotes the conversion of fatty acids to soluble sugars (Figure 9). More importantly, it provides a theoretical and practical basis for exogenous sucrose to enhance oilseed seed vigor.

## Data Availability

All data supporting the findings of this study are available within the paper and within its Supplementary Data, which are published online.

## Supplementary Materials

The following supporting information can be downloaded at: https://www.mdpi.com/article/10.3390/plants14152301/s1, Figure S1. The germination indicator of safflower seeds under different treatments (A) The influence of different aging times on the germination of safflower seeds. (B) The effects of different concentrations of sucrose treatment on the germination of aged safflower seeds. Each symbol represents the average of three replicates ± SE. Different lowercase letters (s) at the top of the bars indicate significant differences between treatment types (*p* < 0.05, LSD). Figure S2. Germination indicator of unaged safflower seeds under exogenous sucrose treatment. Each symbol represents the average of three replicates ± SE. Different lowercase letters (s) at the top of the bars indicate significant differences between treatment types (*p* < 0.05, LSD). Figure S3. Sucrose treatment increased the concentrations of each fatty acid in aged safflower seeds during the imbibition. Safflower seeds of treatment types (seeds without CDT, CDT seeds with H2O, and CDT seeds with sucrose treatment) were employed. (A) The linolic concentration of different treatment types of safflower seeds during imbibition. (B) The oleic concentration of different treatment types of safflower seeds during imbibition. (C) The palmitic concentration of different treatment types of safflower seeds during imbibition. (D) The stearic concentration of different treatment types of safflower seeds during imbibition. Each symbol represents the average of three replicates ± SE. Different lowercase letters (s) at the top of the bars indicate significant differences between treatment types (*p* < 0.05, LSD). Exogenous sucrose at 50 mg·L^−1^ was employed. Figure S4. Sucrose promotes the germination of CDT-aged 15 days safflower seeds (preliminary experiments). Each symbol represents the average of three replicates ± SE. Different lowercase letters (s) next to the data point indicate significant differences between treatment types (*p* < 0.05, LSD). Table S1. The gene expression levels related to DNA replication and base excision repair. Table S2. Gene primers of qPCR.

## References

[1] Zohary D. (2019). Monophyletic vs. polyphyletic origin of the crops on which agriculture was founded in the Near East. Genet. Resour. Crop Evol..

[2] FAO (2020). Faostat/en. https://www.fao.org/faostat/en/#data/QCL.

[3] Sharifi R.S., Namvar A., Sharifi R.S. (2017). Grain filling and fatty acid composition of safflower fertilized with integrated nitrogen fertilizer and biofertilizers. Pesqui. Agropecuária Bras..

[4] Matthaus B., Özcan M.M., Al Juhaimi F.Y. (2015). Fatty acid composition and tocopherol profiles of safflower (*Carthamus tinctorius* L.) seed oils. Nat. Prod. Res..

[5] Amini H., Arzani A., Karami M. (2014). Effect of water deficiency on seed quality and physiological traits of different safflower genotypes. Turk. J. Biol..

[6] Goel A., Goel A.K., Sheoran I.S. (2003). Changes in oxidative stress enzymes during artificial ageing in cotton (*Gossypium hirsutum* L.) seeds. J. Plant Physiol..

[7] Koornneef M., Bentsink L., Hilhorst H. (2022). Seed dormancy and germination. Curr. Opin. Plant Biol..

[8] Bewley J.D., Bradford K.J., Hilhorst H.W.M., Nonogaki H. (2013). Germination. Seeds.

[9] Gran P., Visscher T.W., Bai B., Nijveen H., Mahboubi A., Bakermans L.L., Willems L.A.J., Bentsink L. (2025). Unravelling the dynamics of seed-stored mRNAs during seed priming. New Phytol..

[10] Gu J., Hou D., Li Y., Chao H., Zhang K., Wang H., Xiang J., Raboanatahiry N., Wang B., Li M. (2019). Integration of proteomic and genomic approaches to dissect seed germination vigor in Brassica napus seeds differing in oil content. BMC Plant Biol..

[11] Wang L., Zuo Q., Zheng J., You J., Yang G., Leng S. (2022). Salt stress decreases seed yield and postpones growth process of canola (*Brassica napus* L.) by changing nitrogen and carbon characters. Sci. Rep..

[12] Wei J., Xu L., Shi Y., Cheng T., Tan W., Zhao Y., Li C., Yang X., Ouyang L., Wei M. (2023). Transcriptome profile analysis of Indian mustard (*Brassica juncea* L.) during seed germination reveals the drought stress-induced genes associated with energy, hormone, and phenylpropanoid pathways. Plant Physiol. Biochem..

[13] Ghobadi M.E., Ghobadi M., Zebarjadi A. (2017). Effect of waterlogging at different growth stages on some morphological traits of wheat varieties. Int. J. Biometeorol..

[14] Seneviratne M., Rajakaruna N., Rizwan M., Madawala H.M.S.P., Ok Y.S., Vithanage M. (2019). Heavy metal-induced oxidative stress on seed germination and seedling development: A critical review. Environ. Geochem. Health.

[15] Hura T. (2020). Wheat and barley: Acclimatization to abiotic and biotic stress. Int. J. Mol. Sci..

[16] Rajjou L., Duval M., Gallardo K., Catusse J., Bally J., Job C., Job D. (2012). Seed germination and vigor. Annu. Rev. Plant Biol..

[17] Zhou W., Chen F., Zhao S., Yang C., Meng Y., Shuai H., Luo X., Dai Y., Yin H., Du J. (2019). DA-6 promotes germination and seedling establishment from aged soybean seeds by mediating fatty acid metabolism and glycometabolism. J. Exp. Bot..

[18] Zhang M., Li B., Wan Z., Chen X., Liu C., Liu C., Zhou Y. (2022). Exogenous Spermidine Promotes Germination of Aged Sorghum Seeds by Mediating Sugar Metabolism. Plants.

[19] Theodoulou F.L., Eastmond P.J. (2012). Seed storage oil catabolism: A story of give and take. Curr. Opin. Plant Biol..

[20] Quettier A.L., Shaw E., Eastmond P.J. (2008). SUGAR-DEPENDENT6 encodes a mitochondrial flavin adenine dinucleotide-dependent glycerol-3-p dehydrogenase, which is required for glycerol catabolism and post germinative seedling growth in Arabidopsis. Plant Physiol..

[21] Hurlock A.K., Roston R.L., Wang K., Benning C. (2014). Lipid trafficking in plant cells. Traffic.

[22] Huang Y., Cai S., Ruan X., Xu J., Cao D. (2021). CSN improves seed vigor of aged sunflower seeds by regulating the fatty acid, glycometabolism, and abscisic acid metabolism. J. Adv. Res..

[23] Zhou L., Lu L., Chen C., Zhou T., Wu Q., Wen F., Chen J., Pritchard H.W., Peng C., Pei J. (2022). Comparative changes in sugars and lipids show evidence of a critical node for regeneration in safflower seeds during aging. Front. Plant Sci..

[24] Lv T., Li J., Zhou L., Zhou T., Pritchard H.W., Ren C., Chen J., Yan J., Pei J. (2024). Aging-Induced Reduction in Safflower Seed Germination via Impaired Energy Metabolism and Genetic Integrity Is Partially Restored by Sucrose and DA-6 Treatment. Plants.

[25] Chen C., Wang R., Dong S., Wang J., Ren C.X., Chen C.P., Yan J., Zhou T., Wu Q.H., Pei J. (2022). Integrated proteome and lipidome analysis of naturally aged safflower seeds varying in vitality. Plant Biol..

[26] Li L., Sheen J. (2016). Dynamic and diverse sugar signaling. Curr. Opin. Plant Biol..

[27] Zhu M., Zang Y., Zhang X., Shang S., Xue S., Chen J., Tang X. (2023). Insights into the regulation of energy metabolism during the seed-to-seedling transition in marine angiosperm Zostera marina L.: Integrated metabolomic and transcriptomic analysis. Front. Plant Sci..

[28] Zhou W., Yang Y., Zheng C., Luo X., Chandrasekaran U., Yin H., Chen F., Meng Y., Chen L., Shu K. (2021). Flooding represses soybean seed germination by mediating anaerobic respiration, glycometabolism and phytohormones biosynthesis. Environ. Exp. Bot..

[29] Bhattacharyya S., Sen-Mandi S. (1985). Studies into causes of non-germination of aged wheat seeds. Ann. Bot..

[30] Eastmond P.J., Germain V., Lange P.R., Bryce J.H., Smith S.M., Graham I.A. (2000). Postgerminative growth and lipid catabolism in oilseeds lacking the glyoxylate cycle. Proc. Natl. Acad. Sci. USA.

[31] Borek S., Nuc K. (2011). Sucrose controls storage lipid breakdown on gene expression level in germinating yellow lupine (*Lupinus luteus* L.) seeds. J. Plant Physiol..

[32] Wang B., Zhang Y., Haque M.E., Xu W., Li F., Liu A. (2018). Transcriptomic analyses reveal complex and interconnected sucrose signaling cascades in developing seeds of castor bean. J. Plant Physiol..

[33] Yu J., Lee H., Heo H., Jeong H.S., Sung J., Lee J. (2023). Sucrose-induced abiotic stress improves the phytochemical profiles and bioactivities of mung bean sprouts. Food Chem..

[34] Lara-Núñez A., García-Ayala B.B., Garza-Aguilar S.M., Flores-Sánchez J., Sánchez-Camargo V.A., Bravo-Alberto C.E., Vázquez-Santana S., Vázquez-Ramos J.M. (2017). Glucose and sucrose differentially modify cell proliferation in maize during germination. Plant Physiol. Biochem..

[35] Cui S., Hayashi Y., Otomo M., Mano S., Oikawa K., Hayashi M., Nishimura M. (2016). Sucrose Production Mediated by Lipid Metabolism Suppresses the Physical Interaction of Peroxisomes and Oil Bodies during Germination of Arabidopsis thaliana. J. Biol. Chem..

[36] Galland M., Boutet-Mercey S., Lounifi I., Godin B., Balzergue S., Grandjean O., Morin H., Perreau F., Debeaujon I., Rajjou L. (2014). Compartmentation and dynamics of flavone metabolism in dry and germinated rice seeds. Plant Cell Physiol..

[37] Wang Y., Jiang W., Li C., Wang Z., Lu C., Cheng J., Wei S., Yang J., Yang Q. (2024). Integrated transcriptomic and metabolomic analyses elucidate the mechanism of flavonoid biosynthesis in the regulation of mulberry seed germination under salt stress. BMC Plant Biol..

[38] Kalaivani V., Nikarika R., Shoma N., Arunraj R. (2021). Delayed hydrolysis of Raffinose Family Oligosaccharides (RFO) affects critical germination of chickpeas. 3 Biotech.

[39] Formela-Luboińska M., Chadzinikolau T., Drzewiecka K., Jeleń H., Bocianowski J., Kęsy J., Labudda M., Jeandet P., Morkunas I. (2020). The Role of Sugars in the Regulation of the Level of Endogenous Signaling Molecules during Defense Response of Yellow Lupine to *Fusarium oxysporum*. Int. J. Mol. Sci..

[40] Salam B.B., Barbier F., Danieli R., Teper-Bamnolker P., Ziv C., Spíchal L., Aruchamy K., Shnaider Y., Leibman D., Shaya F. (2021). Sucrose promotes stem branching through cytokinin. Plant Physiol..

[41] Oracz K., Stawska M. (2016). Cellular Recycling of Proteins in Seed Dormancy Alleviation and Germination. Front. Plant Sci..

[42] Galland M., Huguet R., Arc E., Cueff G., Job D., Rajjou L. (2014). Dynamic proteomics emphasizes the importance of selective mRNA translation and protein turnover during Arabidopsis seed germination. Mol. Cell. Proteom..

[43] Huang Y., Mei G., Fu X., Wang Y., Ruan X., Cao D. (2022). Ultrasonic Waves Regulate Antioxidant Defense and Gluconeogenesis to Improve Germination From Naturally Aged Soybean Seeds. Front. Plant Sci..

[44] Hu X., Zhou Q. (2014). Novel hydrated graphene ribbon unexpectedly promotes aged seed germination and root differentiation. Sci. Rep..

[45] Nile S.H., Thiruvengadam M., Wang Y., Samynathan R., Shariati M.A., Rebezov M., Nile A., Sun M., Venkidasamy B., Xiao J. (2022). Nano-priming as emerging seed priming technology for sustainable agriculture-recent developments and future perspectives. J. Nanobiotechnol..

[46] Li Y., Liang L., Li W., Ashraf U., Ma L., Tang X., Pan S., Tian H., Mo Z. (2021). ZnO nanoparticle-based seed priming modulates early growth and enhances physio-biochemical and metabolic profiles of fragrant rice against cadmium toxicity. J. Nanobiotechnol..

[47] Gupta N., Singh P.M., Sagar V., Pandya A., Chinnappa M., Kumar R., Bahadur A. (2022). Seed Priming with ZnO and Fe_3_O_4_ Nanoparticles Alleviate the Lead Toxicity in *Basella alba* L. through Reduced Lead Uptake and Regulation of ROS. Plants.

[48] Basnet R.K., Del Carpio D.P., Xiao D., Bucher J., Jin M., Boyle K., Fobert P., Visser R.G., Maliepaard C., Bonnema G. (2016). A Systems Genetics Approach Identifies Gene Regulatory Networks Associated with Fatty Acid Composition in *Brassica rapa* Seed. Plant Physiol..

[49] Faraoni P., Sereni E., Gnerucci A., Cialdai F., Monici M., Ranaldi F. (2019). Glyoxylate cycle activity in *Pinus pinea* seeds during germination in altered gravity conditions. Plant Physiol. Biochem..

[50] Adham A.R., Zolman B.K., Millius A., Bartel B. (2005). Mutations in Arabidopsis acyl-CoA oxidase genes reveal distinct and overlapping roles in beta-oxidation. Plant J..

[51] Eastmond P.J., Graham I.A. (2000). The multifunctional protein AtMFP2 is co-ordinately expressed with other genes of fatty acid beta-oxidation during seed germination in *Arabidopsis thaliana* (L.). Heynh. Biochem. Soc. Trans..

[52] Li Y., Liu Y., Zolman B.K. (2019). Metabolic Alterations in the Enoyl-CoA Hydratase 2 Mutant Disrupt Peroxisomal Pathways in Seedlings. Plant Physiol..

[53] Delker C., Zolman B.K., Miersch O., Wasternack C. (2007). Jasmonate biosynthesis in *Arabidopsis thaliana* requires peroxisomal beta-oxidation enzymes--additional proof by properties of pex6 and aim1. Phytochemistry.

[54] Pinfield-Wells H., Rylott E.L., Gilday A.D., Graham S., Job K., Larson T.R., Graham I.A. (2005). Sucrose rescues seedling establishment but not germination of Arabidopsis mutants disrupted in peroxisomal fatty acid catabolism. Plant J..

[55] Rylott E.L., Eastmond P.J., Gilday A.D., Slocombe S.P., Larson T.R., Baker A., Graham I.A. (2006). The *Arabidopsis thaliana* multifunctional protein gene (MFP2) of peroxisomal beta-oxidation is essential for seedling establishment. Plant J..

[56] Domergue J.B., Abadie C., Limami A., Way D., Tcherkez G. (2019). Seed quality and carbon primary metabolism. Plant Cell Environ..

[57] Graham I.A. (2008). Seed storage oil mobilization. Annu. Rev. Plant Biol..

[58] Nigam M., Mishra A.P., Salehi B., Kumar M., Sahrifi-Rad M., Coviello E., Iriti M., Sharifi-Rad J. (2019). Accelerated ageing induces physiological and biochemical changes in tomato seeds involving MAPK pathways. Sci. Hortic..

[59] Zhang W., Mace W.J., Matthew C., Card S.D. (2019). The Impact of Endophyte Infection, Seed Aging, and Imbibition on Selected Sugar Metabolite Concentrations in Seed. J. Agric. Food Chem..

[60] Bernal-Lugo I., Leopold A.C. (1992). Changes in Soluble Carbohydrates during Seed Storage. Plant Physiol..

[61] Pereira Lima J.J., Buitink J., Lalanne D., Rossi R.F., Pelletier S., da Silva E.A.A., Leprince O. (2017). Molecular characterization of the acquisition of longevity during seed maturation in soybean. PLoS ONE.

[62] Li W., Huang L., Liu N., Pandey M.K., Chen Y., Cheng L., Guo J., Yu B., Luo H., Zhou X. (2021). Key Regulators of Sucrose Metabolism Identified through Comprehensive Comparative Transcriptome Analysis in Peanuts. Int. J. Mol. Sci..

[63] Eastmond P.J., Astley H.M., Parsley K., Aubry S., Williams B.P., Menard G.N., Craddock C.P., Nunes-Nesi A., Fernie A.R., Hibberd J.M. (2015). Arabidopsis uses two gluconeogenic gateways for organic acids to fuel seedling establishment. Nat. Commun..

[64] Silva M.F.D., Soares J.M., Xavier W.A., Silva F.C.D.S., Silva F.L.D., Silva L.J.D. (2023). The role of the biochemical composition of soybean seeds in the tolerance to deterioration under natural and artificial aging. Heliyon.

[65] Diantina S., McGill C., Millner J., Nadarajan J., Pritchard H.W., Colville L., Clavijo McCormick A. (2022). Seed viability and fatty acid profiles of five orchid species before and after ageing. Plant Biol..

[66] Kozuka T., Sawada Y., Imai H., Kanai M., Hirai M.Y., Mano S., Uemura M., Nishimura M., Kusaba M., Nagatani A. (2020). Regulation of Sugar and Storage Oil Metabolism by Phytochrome during De-etiolation. Plant Physiol..

[67] Hayashi Y., Sato N., Shinozaki A., Watanabe M. (2015). Increase in peroxisome number and the gene expression of putative glyoxysomal enzymes in Chlamydomonas cells supplemented with acetate. J. Plant Res..

[68] Zhao S., Garcia D., Zhao Y., Huang D. (2021). Hydro-Electro Hybrid Priming Promotes Carrot (*Daucus carota* L.) Seed Germination by Activating Lipid Utilization and Respiratory Metabolism. Int. J. Mol. Sci..

[69] Wu W.L., Hsiao Y.Y., Lu H.C., Liang C.K., Fu C.H., Huang T.H., Chuang M.H., Chen L.J., Liu Z.J., Tsai W.C. (2020). Expression regulation of MALATE SYNTHASE involved in glyoxylate cycle during protocorm development in Phalaenopsis aphrodite (Orchidaceae). Sci. Rep..

[70] Ma Z., Marsolais F., Bernards M.A., Sumarah M.W., Bykova N.V., Igamberdiev A.U. (2016). Glyoxylate cycle and metabolism of organic acids in the scutellum of barley seeds during germination. Plant Sci..

[71] Eastmond P.J. (2006). SUGAR-DEPENDENT1 encodes a patatin domain triacylglycerol lipase that initiates storage oil breakdown in germinating Arabidopsis seeds. Plant Cell.

[72] Campobenedetto C., Grange E., Mannino G., van Arkel J., Beekwilder J., Karlova R., Garabello C., Contartese V., Bertea C.M. (2020). A Biostimulant Seed Treatment Improved Heat Stress Tolerance During Cucumber Seed Germination by Acting on the Antioxidant System and Glyoxylate Cycle. Front. Plant Sci..

[73] Song Y., Gao X., Wu Y. (2021). Key Metabolite Differences Between Korean Pine (*Pinus koraiensis*) Seeds With Primary Physiological Dormancy and No-Dormancy. Front. Plant Sci..

[74] He Y., Cheng J., He Y., Yang B., Cheng Y., Yang C., Zhang H., Wang Z. (2019). Influence of isopropylmalate synthase OsIPMS1 on seed vigour associated with amino acid and energy metabolism in rice. Plant Biotechnol. J..

[75] Yang B., Chen M., Zhan C., Liu K., Cheng Y., Xie T., Zhu P., He Y., Zeng P., Tang H. (2022). Identification of OsPK5 involved in rice glycolytic metabolism and GA/ABA balance for improving seed germination via genome-wide association study. J. Exp. Bot..

[76] Guo Z., Zhao J., Wang M., Song S., Xia Z. (2021). Sulfur dioxide promotes seed germination by modulating reactive oxygen species production in maize. Plant Sci..

[77] Lei K., Sun S., Zhong K., Li S., Hu H., Sun C., Zheng Q., Tian Z., Dai T., Sun J. (2021). Seed soaking with melatonin promotes seed germination under chromium stress via enhancing reserve mobilization and antioxidant metabolism in wheat. Ecotoxicol. Environ. Saf..

[78] Chen L., Lu B., Liu L., Duan W., Jiang D., Li J., Zhang K., Sun H., Zhang Y., Li C. (2021). Melatonin promotes seed germination under salt stress by regulating ABA and GA3 in cotton (*Gossypium hirsutum* L.). Plant Physiol. Biochem..

[79] Ventura L., Donà M., Macovei A., Carbonera D., Buttafava A., Mondoni A., Rossi G., Balestrazzi A. (2012). Understanding the molecular pathways associated with seed vigor. Plant Physiol. Biochem..

[80] Kiran K.R., Deepika V.B., Swathy P.S., Prasad K., Kabekkodu S.P., Murali T.S., Satyamoorthy K., Muthusamy A. (2020). ROS-dependent DNA damage and repair during germination of NaCl primed seeds. J. Photochem. Photobiol. B.

[81] Forti C., Shankar A., Singh A., Balestrazzi A., Prasad V., Macovei A. (2020). Hydropriming and Biopriming Improve Medicago truncatula Seed Germination and Upregulate DNA Repair and Antioxidant Genes. Genes.

[82] Millennium Seed Bank Partnership (2022). Germination Testing: Procedures and Evaluation. https://brahmsonline.kew.org/Content/Projects/msbp/resources/Training/13a-Germination-testing-procedures.pdf.

[83] Zhou T., Qiu X., Zhao L., Yang W., Wen F., Wu Q., Yan J., Xu B., Chen J., Ma Y. (2022). Optimal light intensity and quality increased the saffron daughter corm yield by inhibiting the degradation of reserves in mother corms during the reproductive stage. Ind. Crops Prod..

[84] Yang C., Iqbal N., Hu B., Zhang Q., Wu H., Liu X., Zhang J., Liu W., Yang W., Liu J. (2017). Targeted metabonomics analysis of fatty acid in soybean seed by GC-MS reveal the metabolic manipulation of shading in intercropping system. Anal. Methods.

[85] Wu Z., Liu H., Zhan W., Yu Z., Qin E., Liu S., Yang T., Xiang N., Kudrna D., Chen Y. (2021). The chromosome-scale reference genome of safflower (*Carthamus tinctorius* L.) provides insights into linoleic acid and flavonoid biosynthesis. Plant Biotechnol. J..

[86] Livak K.J., Schmittgen T.D. (2001). Analysis of relative gene expression data using real-time quantitative PCR and the 2^−ΔΔCT^ method. Methods.

