# Supplementary material for: Exogenous Sucrose Improves the Vigor of Aged Safflower Seeds by Mediating Fatty Acid Metabolism and Glycometabolism
Source: Plants (Basel). 2025 Jul 25;14(15):2301. doi: 10.3390/plants14152301 (PMC12348142; doi:10.3390/plants14152301)
Supplement: Supplementary file 1 [file plants-14-02301-s001.zip › plants-3733069-supplementary.pdf]

Supplementary Material

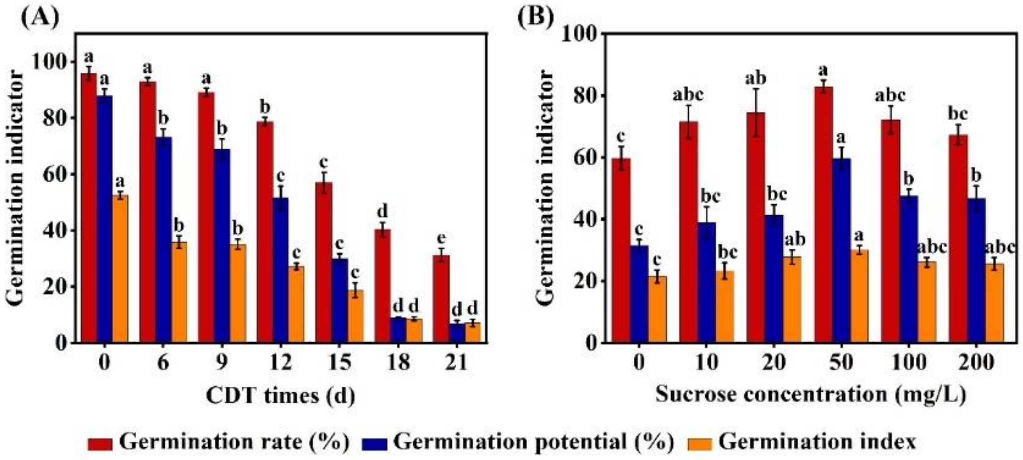

Figure S1 The germination indicator of safflower seeds under different treatments (A) The influence of different aging times on the germination of safflower seeds. (B) The effects of different concentrations of sucrose treatment on the germination of aged safflower seeds. Each symbol represents the average of three replicates ± SE. Different lowercase letters (s) at the top of the bars indicate significant differences between treatment types ($P < 0.05$, LSD).

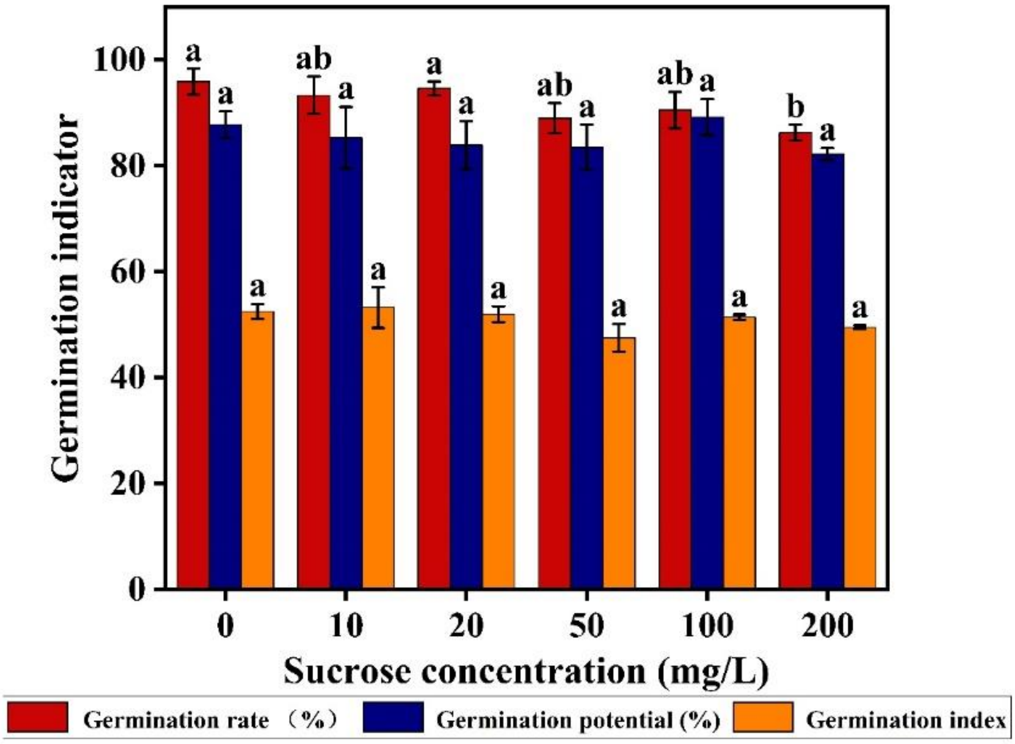

Figure S2 Germination indicator of unaged safflower seeds under exogenous sucrose treatment. Each symbol represents the average of three replicates ± SE. Different lowercase letters (s) at the top of the bars indicate significant differences between treatment types ($P < 0.05$, LSD).

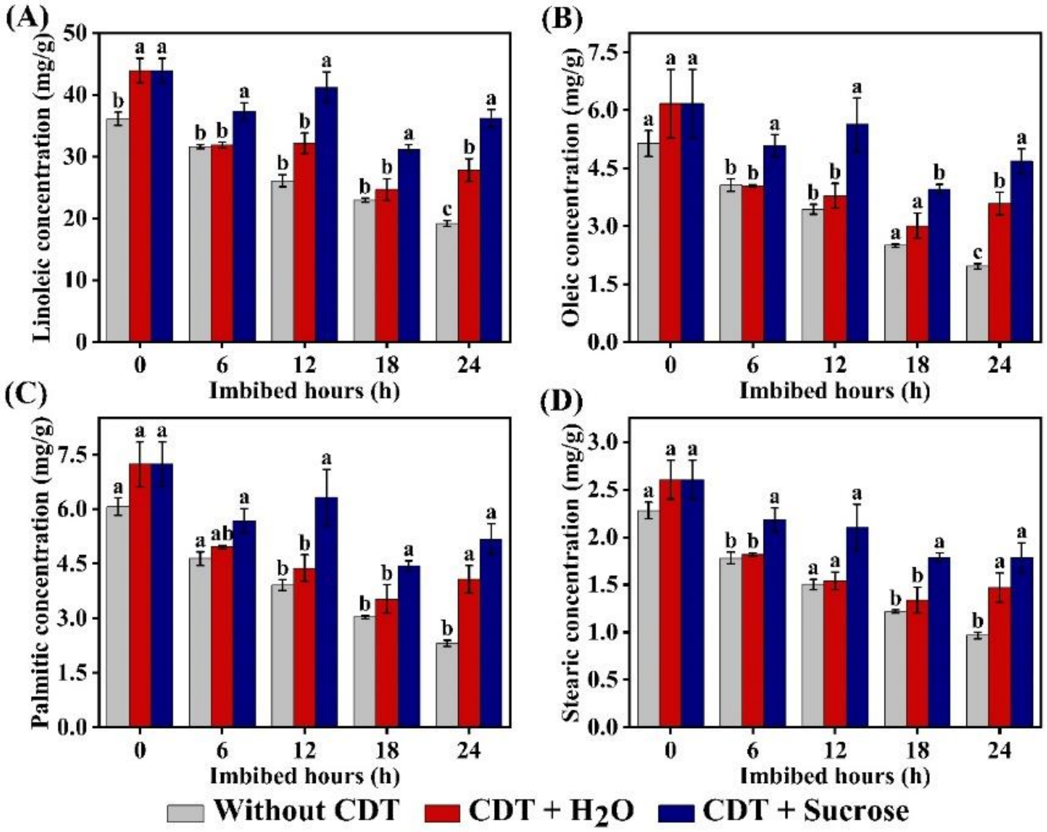

Figure S3 Sucrose treatment increased the concentrations of each fatty acid in aged safflower seeds during the imbibition. Safflower seeds of treatment types (seeds without CDT, CDT seeds with H_2O, and CDT seeds with sucrose treatment) were employed. (A) The linolic concentration of different treatment types of safflower seeds during imbibition. (B) The oleic concentration of different treatment types of safflower seeds during imbibition. (C) The palmitic concentration of different treatment types of safflower seeds during imbibition. (D) The stearic concentration of different treatment types of safflower seeds during imbibition. Each symbol represents the average of three replicates ± SE. Different lowercase letters (s) at the top of the bars indicate significant differences between treatment types ($P < 0.05$, LSD). Exogenous sucrose at 50 $mg \cdot L^{-1}$ was employed.

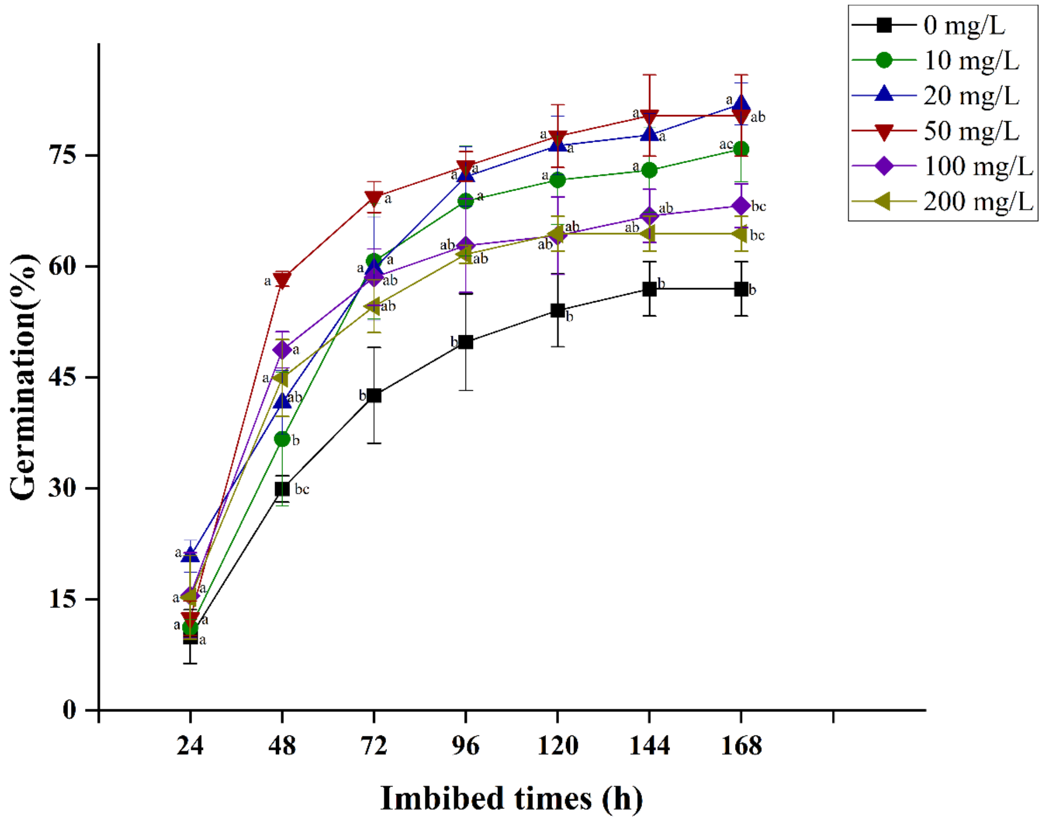

Figure S4 Sucrose promotes the germination of CDT-aged 15 days safflower seeds (preliminary experiments). Each symbol represents the average of three replicates ± SE. Different lowercase letters (s) next to the data point indicate significant differences between treatment types ($P < 0.05$, LSD).

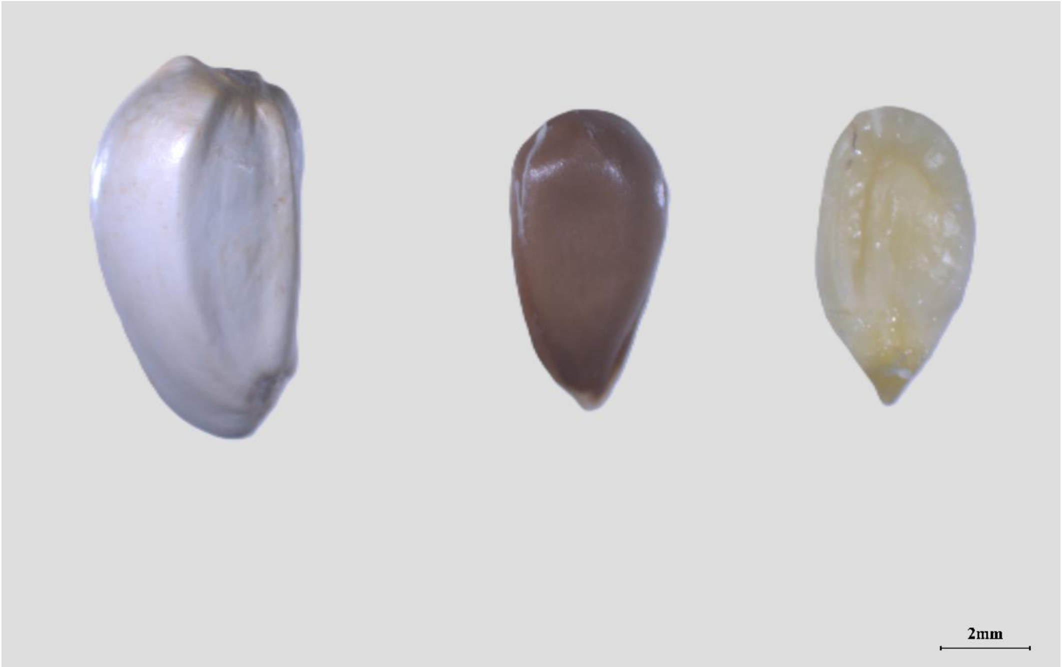

Figure S5 Microscope image of red flower seeds (from left to right: dry seeds, shelled seeds, and cross-section)

Table S1 The gene expression levels related to DNA replication and base excision repair

Gene ID	Log_2Fold Change	Pvalue	Padj	Pathway
CtAH04G0202600	1.491692254	$1.53*10^{-23}$	$3.84*10^{-22}$	DNA replication
CtAH10G0038800	1.443173973	$1.61*10^{-20}$	$3.10*10^{-19}$	DNA replication
CtAH08G0099700	1.215340995	$6.70*10^{-18}$	$1.03*10^{-16}$	DNA replication
CtAH06G0053400	1.189553409	$2.92*10^{-17}$	$4.26*10^{-16}$	DNA replication
CtAH09G0144300	1.140283784	$4.01*10^{-17}$	$5.76*10^{-16}$	DNA replication
CtAH08G0273000	1.563441263	$5.29*10^{-17}$	$7.52*10^{-16}$	DNA replication
CtAH01G0210000	1.338872932	$3.63*10^{-13}$	$3.52*10^{-12}$	DNA replication
CtAH10G0243300	-1.185973243	$9.60*10^{-13}$	$8.95*10^{-12}$	DNA replication
CtAH03G0033800	1.023698231	$7.31*10^{-11}$	$5.53*10^{-10}$	DNA replication
CtAH02G0086200	1.042941217	$2.93*10^{-10}$	$2.05*10^{-9}$	DNA replication
CtAH09G0104200	1.141880661	$2.79*10^{-6}$	$1.21*10^{-5}$	DNA replication
CtAH11G0172200	-1.259236249	$1.31*10^{-3}$	$3.72*10^{-3}$	DNA replication
CtAH11G0083700	-1.080270611	$1.37*10^{-27}$	$4.86*10^{-26}$	Base excision repair
novel.2111	-1.260119309	$1.27*10^{-16}$	$1.74*10^{-15}$	Base excision repair
CtAH01G0050800	1.158262403	$2.97*10^{-14}$	$3.21*10^{-13}$	Base excision repair
CtAH12G0097900	1.293447242	$1.04*10^{-12}$	$9.66*10^{-12}$	Base excision repair
CtAH06G0265600	1.345444662	$4.29*10^{-9}$	$2.66*10^{-8}$	Base excision repair
CtAH09G0237400	1.593813216	$2.84*10^{-8}$	$1.60*10^{-7}$	Base excision repair
CtAH10G0098600	-1.938725689	$4.08*10^{-7}$	$1.98*10^{-6}$	Base excision repair
CtAH09G0083800	1.230403511	$2.04*10^{-5}$	$7.85*10^{-5}$	Base excision repair
CtAH01G0101400	1.831444891	$5.16*10^{-4}$	$1.58*10^{-3}$	Base excision repair
CtAH08G0212100	3.136922829	$7.14*10^{-4}$	$2.14*10^{-3}$	Base excision repair
CtAH11G0180200	-1.259236249	$7.48*10^{-3}$	$1.80*10^{-2}$	Base excision repair

Table S2 Gene primers of qPCR

Number	Gene ID	Primer	For（5’-3’）	Length(bp)
1	CtAH06G0067600	PK1F	ACACCATCTTGTGTGCCGAT	20
2	CtAH06G0067600	PK1R	ACGAGCAACCATGAACGAGT	20
3	CtAH08G0152700	PCK1F	ATACTCGTGCAGCATACCCG	20
4	CtAH08G0152700	PCK1R	ACAAGCCATCCAGTAGCACC	20

5	CtAH10G0123800	SDP1F	AAGAATGGGCTCATGCTGCT	20
6	CtAH10G0123800	SDP1R	CAGATAATGGATCCGGCGCT	20
7	CtAH03G0131700	ACOX2F	ACAAAACACTTCCGGGGGTT	20
8	CtAH03G0131700	ACOX2R	GGCTTCTTCGGAGGACCAAA	20
9	CtAH03G0294000	MFPAF	CCGATGGGGTGGCTATCATC	20
10	CtAH03G0294000	MFPAR	GTCGACATCGCTTTGCCATC	20
11	CtAH09G0237400	PARP1F	AGCGAGTGAAGAGGCGAAAA	20
12	CtAH09G0237400	PARP1R	CCCACTTCCGGAACACATGA	20
13	CtAH12G0162000	PCNAF	ACGCCAATTTCGATTGCTCG	20
14	CtAH12G0162000	PCNAR	CCATCATCGGCCTTGAGAGT	20
15	CtAH01G0004900	G6PDF	GCCACTATGGAACCGTGACA	20
16	CtAH01G0004900	G6PDR	ACCATAGTTGCGAAGGTGGG	20
17	CtAH06G0053400	DNLI1F	GATGCACTCATGTCCAACGC	20
18	CtAH06G0053400	DNLI1R	AGCAAATCATCGGGTGTCGT	20
19	CtAH01G0004900	GPDA2F	TCTCGTTCATGGCGGAACAA	20
20	CtAH01G0004900	GPDA2R	CTGGTACAGCATGGAGGCAA	20
